# Successful **a**uxiliary two-**s**taged **p**art**i**al **re**section liver transplantation (**ASPIRE**-LTx) for end-stage liver disease to avoid small-for-size situations

**DOI:** 10.1186/s12893-021-01167-6

**Published:** 2021-03-26

**Authors:** Stefan M. Brunner, Frank W. Brennfleck, Henrik Junger, Jirka Grosse, Birgit Knoppke, Edward K. Geissler, Michael Melter, Hans J. Schlitt

**Affiliations:** 1 Department of Surgery, University Medical Center Regensburg, Franz-Josef-Strauss-Allee 11, 93053 Regensburg, Germany; 2 University Children’s Hospital Regensburg (KUNO), University Medical Center Regensburg, Regensburg, Germany; 3 Department of Nuclear Medicine, University Medical Center Regensburg, Regensburg, Germany

**Keywords:** Left liver living donation, Split liver transplantation, Auxiliary liver transplantation, ASPIRE

## Abstract

**Background:**

Risks for living-liver donors are lower in case of a left liver donation, however, due to lower graft volume, the risk for small-for-size situations in the recipients increases. This study aims to prevent small-for-size situations in recipients using an auxiliary two-staged partial resection liver transplantation (LTX) of living-donated left liver lobes.

**Case presentation:**

Two patients received a two-stage auxiliary LTX using living-donated left liver lobes after left lateral liver resection. The native extended right liver was removed in a second operation after sufficient hypertrophy of the left liver graft had occurred. Neither donor developed postoperative complications. In both recipients, the graft volume increased by an average of 105% (329 ml to 641 ml), from a graft-to-body-weight ratio of 0.54 to 1.08 within 11 days after LTX, so that the remnant native right liver could be removed. No recipient developed small-for-size syndrome; graft function and overall condition is good in both recipients after a follow-up time of 25 months.

**Conclusions:**

Auxiliary two-staged partial resection LTX using living-donor left lobes is technically feasible and can prevent small-for-size situation. This new technique can expand the potential living-donor pool and contributes to increase donor safety.

## Background

For some patients living-donor liver transplantation (LTX) remains the only treatment option for end-stage liver disease [1]. Donor safety must be considered in this setting [2]. One option to increase donor safety in adult-to-adult living donor LTX is using only either the left or left-lateral liver lobe for transplantation. In this case, risks for recipients significantly increase, mostly due to an increased chance for small-for-size syndrome and hepatic artery thrombosis which are associated with a graft-to-body-weight ratio < 0.6%, resulting in 1 year graft survival rates < 60% [2]. To overcome this problem, we aimed to investigate the safety and feasibility of liver hypertrophy techniques known from ALLPS procedure combined with an auxiliary LTX in a two-stage concept, which is technically demanding, but shifts potential risks from the donor towards the recipient [3].

## Case presentation

### Methods

In the donors, a standard left liver resection (segment I, II, IV) using crush-clamp technique with bipolar sealing and clipping of larger structures was performed. The left hepatic artery, the portal vein and middle and left hepatic vein were sutured and cut. The graft was flushed with heparin immediately and perfused with HTK solution via the portal vein.

In the recipients, after liver mobilization and cholecystectomy, the liver hilum was dissected, and the main portal vein and left liver artery were isolated; then, the left-lateral liver was resected. The left portal vein and bile duct were sutured and cut, and the left hepatic vein clamped. The vena cava was dissected around the left hepatic vein orifice, so that an incision could be made from the left vein into the vena cava after clamping to enable a wide venous anastomosis.

In the graft, the left and middle donor veins were sutured together to receive a common ostium. The venous and subsequent portal anastomosis (E/E) was performed. After reperfusion, the donor artery was anastomosed onto the left recipient liver artery (E/E). The biliodigestive anastomosis was performed during the planned relaparotomy. During hepatectomy completion, the liver was mobilized from the vena cava, and the right hilar structures and right and middle hepatic veins were dissected and cut between sutures.

Donors and recipients were evaluated using CT and MRI scans with arterial and portovenous contrast phases and additionally MRCP scans for evaluation of bile duct anatomy. Preoperative volumes for left and right liver lobes and whole liver after hypertrophy at postoperative day 10–12 were calculated from CT scans. Furthermore, ^99m^Tc-mebrofenin hepatobiliary scintigraphy (HBS) scans were performed at postoperative day 1 and 10 to monitor future graft function, and liver perfusion was assessed by duplex and contrast enhanced ultrasound [4–6].

### Case 1

**Fig. 1:**
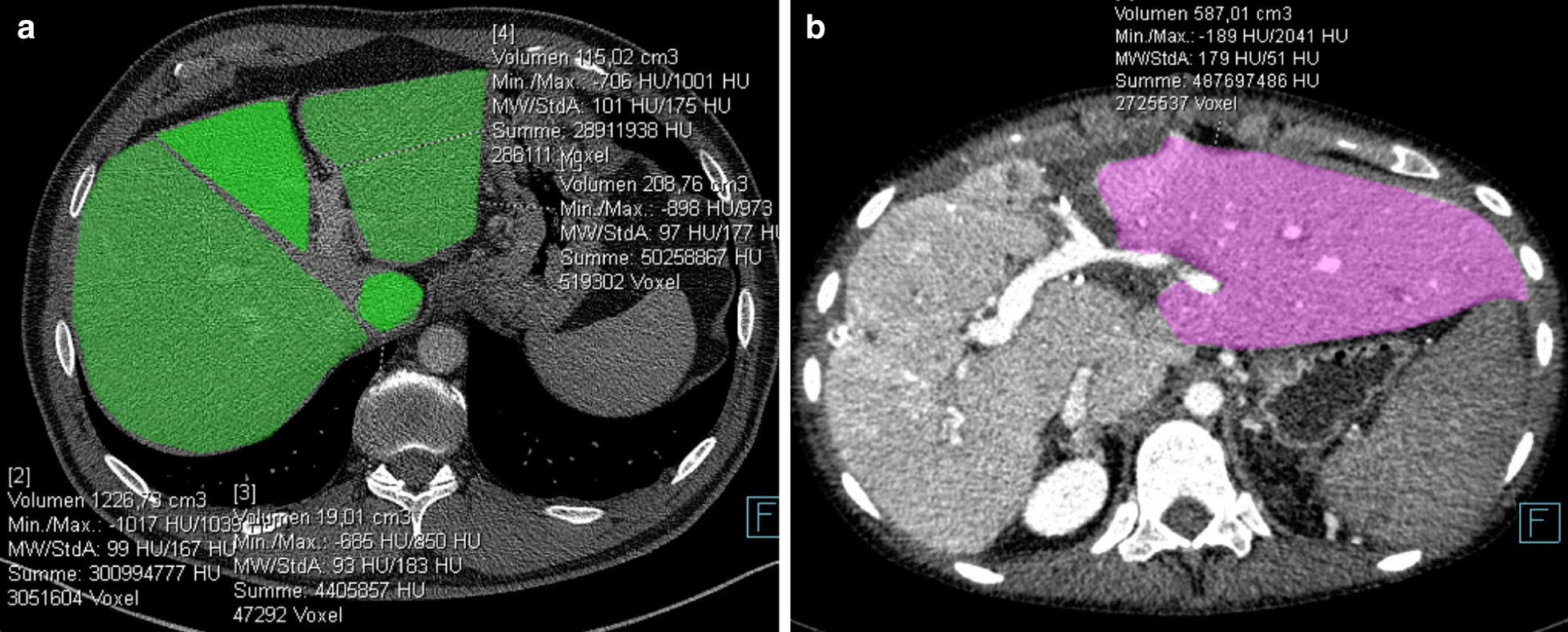
Preoperative CT scan of donor 1. **a** Volumetry of liver segment II/III (209 cm^3^), segment IV (115 cm^3^), segment I (19 cm^3^) and segments V–VIII (1227 cm^3^). **b** CT scan of recipient 1 at postoperative day 12 (before removal of the native right liver) with volumetry of the graft (587 cm^3^) after hypertrophy

Recipient 1, a 30 years-old female with overlap syndrome of autoimmune hepatitis Type 1, primary sclerosing cholangitis and severe portal hypertension, received a 324 cm^3^ graft (graft-to-body-weight ratio 0.65%; Fig. 1a) with two arteries, one for liver segment IV from the standard left artery and one accessory left for segments II/III; these arteries were reconstructed into one anastomosis. The portal vein was anastomosed to the distal left portal vein (E/E to a segment III branch). Since portal vein pressure measurement detected hyperperfusion (18–20 mmHg), the splenic artery was ligated, which reduced portovenous pressure (12 mmHg). Due to instable positioning, the liver was packed and biliodigestive anastomosis was not directly performed. Two days later during the next look operation, a biliodigestive anastomosis was performed with an Roux-en-Y jejunal loop. After graft hypertrophy (81%; Figs. 1b, 3), the patient was reoperated at postoperative day (POD) 14 and the remaining native right liver including segment one was resected. Due to traction during this operation, the biliodigestive anastomosis became dehiscent and was reanastomosed. Due to reduced arterial perfusion in segment II/III in duplex ultrasound controls and a stenosis at the arterial anastomosis (CT scan), the arterial anastomosis was dilated angiographically on POD 17. Since the patient developed 2 l ascites per day beginning at POD 20, another CT scan was performed showing a kinking and stenosis of the left hepatic venous anastomosis, possibly due to liver hyperthrophy and positioning. Consequently, angioplasty with left liver vein stenting was necessary; thereafter, aspirin 100 mg/day was administered. Six weeks after LTX, remaining drains showed biliary secretion so that a PTCD was placed which showed a partial biliary leakage at the biliodigestive anastomosis; the leakage stopped 6 days after PTCD treatment. Consecutively, ascites production decreased, all drains could be removed, and the patient was discharged in good condition 8 weeks after LTX; the PTCD was removed 3 months post-implantation.

One year after LTX an anastomotic portal vein stenosis developed, with the patient receiving a transhepatic angioplasty and portal vein stent (uncovered, self-expanding) during a 1-day hospital admission. Until now (2½ years post-LTX) liver function has been good (normal laboratory values) and the patient is back to work and taking care of her child.

### Case 2

**Fig. 2:**
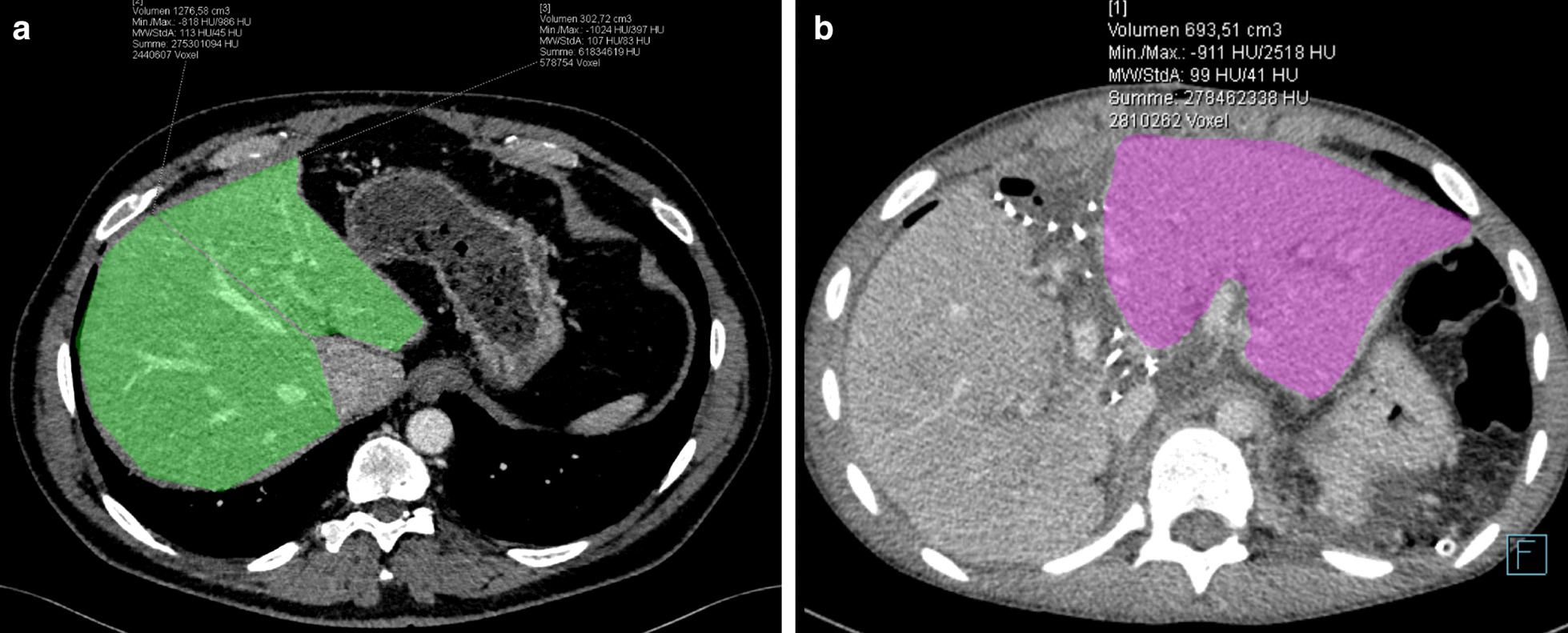
Preoperative CT scan of donor 2. **a** Volumetry of liver segment II/III (303 cm^3^) and segments V–VIII (1277 cm^3^). **b** CT scan of recipient 2 at postoperative day 10 (before removal of the native right liver) with volumetry of the graft (694 cm^3^) after hypertrophy

**Fig. 3:**
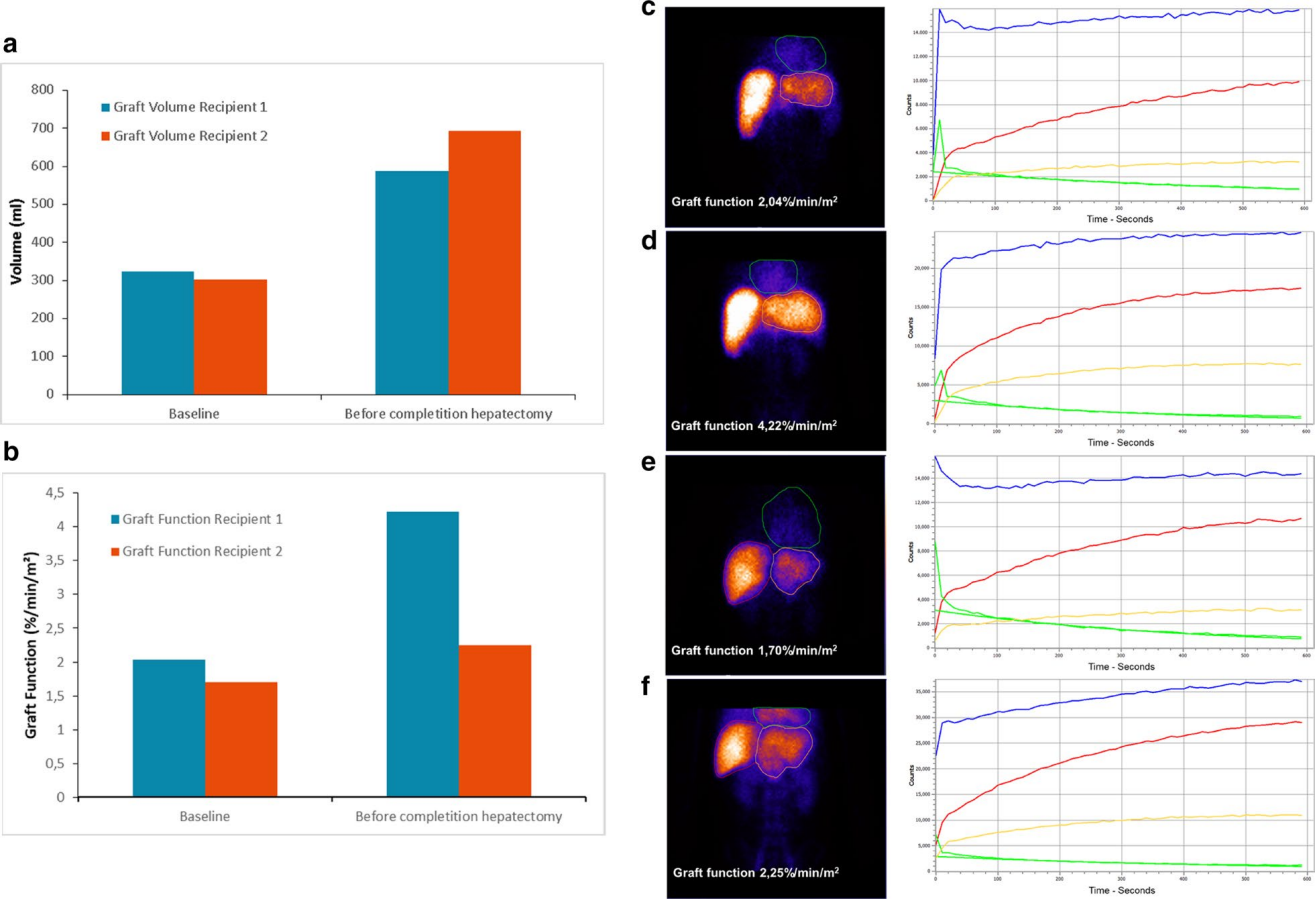
Postoperative course of recipients` graft volume and function. Development of **a** graft volume and **b** graft function calculated from hepatobiliary scintigraphy (HBS) at baseline (transplantation) and before completion hepatectomy. Summed HBS images after i.v. injection of ^99m^Tc-mebrofenin and corresponding time-activity curve of **c** recipient 1 at transplantation and **d** before removal of the native right liver and **e** recipient 2 at transplantation and **f** before removal of the native right liver. Regions of interest were drawn around the native liver and liver graft (red line), the liver graft alone (yellow line) and the heart defined as blood pool (green line). The second green line represents the blood pool fit; the blue line shows the total counts

Recipient 2, a 16 year-old male with congenital liver fibrosis, consecutive cirrhosis and severe portal hypertension, received a 303 cm^3^ graft (graft-to-body-weight ratio 0.43%; Fig. 2a) also with two separately anastomosed arteries. The portal vein was anastomosed E/S to the portal vein main stem. Due to portal hypertension and splenomegaly, a simultaneous splenectomy was performed. The biliodigestive anastomosis was not performed during the first operation (bile duct drained externally via a small luminal bile duct catheter) with local packing. During the planned second look operation at POD 1, a portal vein thrombosis was detected without kinking or detection of anastomotic problems. The thrombus was surgically removed through a portal vein incision, with a catheter placed for 48 h Alteplase lysis therapy into a mesenteric vein branch; no further sign of thrombosis were detected by duplex ultrasound controls. This catheter was removed at POD 3 and a biliodigestive anastomosis was sutured using an Rouy-en-Y jejunal loop. After graft hypertrophy (129%; Figs. 2b, 3), the remaining native liver was removed without complications at POD 10.

Recipient 2 had an uneventful course after discharge (5 weeks after LTX), with a mild cellular rejection episode 1 year after LTX. Duplex ultrasounds showed normal liver graft perfusion and no signs of fibrosis. Liver function is good and the patient currently attends school now 20 months post-LTX.

Both donors had an uncomplicated intra- and postoperative course, were discharged in good condition at postoperative day 7–8, and had no medical problems following living donation.

## Discussion and conclusions

We describe a new treatment concept for patients with end-stage liver disease in whom a living-donor left liver lobe is used in an auxiliary two-stage LTX setting to avoid a potential small-for-size situation. Our approach uses a left liver lobe instead of a left-lateral lobe in an auxiliary setting in cirrhotic patients. Here, we show the method is feasible and can be performed safely and successfully, at least in the first two patients. This is in contrast to a study that also described a treatment concept for cirrhosis where two recipients after left-lateral liver resection then received auxillary LTX using left-lateral grafts [7]. After graft hypertrophy, in one patient the procedure was completed by resection of the native liver, but the other patient required retransplantation [7].

Similar approaches were undertaken in the treatment of irresectable colorectal liver metastases but for non-cirrhotic livers. In this setting, after left hepatectomy, a right portal vein ligation was performed, followed by transplantation of a living-donated left-lateral liver lobe as auxiliary partial orthotopic LTX followed by completion hepatectomy 2 weeks later [8]. In contrast to our study, this concept is performed in a non-cirrhotic liver [8, 9]. This enables the surgeon to perform the necessary left hepatectomy and, additionally, a right portal vein ligation to enhance graft hypertrophy. In our study, the recipients already suffered from severe portal hypertension with massive splenomegaly (invasively measured portovenous pressure 18–20 mmHg). Therefore, it was not necessary and possible to ligate the right portal vein. In contrast, we were even forced, to reduce portal vein pressure.

Generally, management of portal vein perfusion is important to prevent small-for-size-syndrome with rather small liver grafts [10, 11]. Hemiportocaval shunt is one possible solution, splenectomy or splenic artery ligation are others [11–13]. In the situation of a temporarily remaining native right liver where a hemiportocaval shunt is complicated, we decided for splenectomy in one recipient and splenic artery ligation in the other, which is also known to be an effective method to avoid portal hyperperfusion and was successful in our recipients (portovenous pressure 6–8 mm Hg) [12, 13].

Multiple complications occurred in these cases. We had to deal with arterial stenosis, portal vein thrombosis, biliary leakage, venous outflow obstruction due to graft hypertrophy and portal vein stenosis in the long-term follow-up. If complications do occur, “hypersmall” left-lateral liver grafts as used in one of the described studies are at high risk of liver failure due to lacking functional reserve [3]. Instead, we used left liver lobes (liver segments II/III/IV) with slightly higher graft volume. This graft-size optimization strategy is a reasonable explanation why our recipients could safely undergo completion hepatectomy after an average of 12 days, facilitating long-term graft and patient survival in spite of several complications.

Decide on timing of completion hepatectomy is difficult. For this decision we used CT-volumetric measurements and hepatobiliary scintigraphy together with regular laboratory parameters. While CT volumetry is an established method to quantify liver hypertrophy, hepatobiliary scintigraphy adds a functional parameter to simple volume measurement, but has not been routinely used in the setting of LTX or liver hyperthrophy. We suggest that this method needs to be critically evaluated further. While, in principal, the longer it is possible to wait, the better the liver hypertrophy will be, this must be balanced against potential increasing surgical problems regarding adhesions and reduced intraabdominal space. After this two case experience, we consider 10–12 days after the initial operative step as the best timing for completing hepatectomy with sufficient time for graft hypertrophy [3].

Taking our complications into consideration—after this two case experience—our “optimized” technical approach would first be to anastomose the portal vein to the recipients’ main portal trunk, since this reconstruction is more stable with regard to positioning of the liver and not overly long after removal of the native liver. Second, we suggest resecting liver segment I in the recipient during the first operative step, providing an easier approach during the completion operation resulting in less manipulation of the transplanted graft. Third, we suggest a second look operation at POD 1 and delay of the biliodigestive anastomosis to this time point; this approach is meant to increase patient and graft safety by directly evaluating the graft, with assurance of optimal blood in- and outflow. Moreover, this step provides an important opportunity to further position the graft, which can change dramatically after completion hepatectomy.

In conclusion, these cases demonstrate that living-donor left lobe auxiliary two-stage LTX to avoid small-for-size situation in adults/adolescents is safe and technically feasible. This new technique could expand the potential living-donor pool, and in selected recipient/donor combinations enables a living liver donation and additionally contributes to increase donor safety.

## Data Availability

The datasets used and/or analysed during the current study are available from the corresponding author on reasonable request.

## Abbreviations

LTX: Liver transplantation
ALLPS: Associating liver partition and portal vein ligation for staged hepatectomy
HTK: Histidine-tryptophan-ketoglutarate
E/E: End-to-end
HBS: Hepatobiliary scintigraphy
POD: Postoperative day
CT: Computer tomography
PTCD: Percutaneous transhepatic cholangio-drainage
E/S: End-to-side

## References

[1] Kling CE, Perkins JD, Reyes JD, Montenovo MI (2019). Living donation versus donation after circulatory death liver transplantation for low model for end-stage liver disease recipients. Liver Transpl.

[2] Sánchez-Cabús S, Cherqui D, Rashidian N, Pittau G, Elkrief L, Vanlander A (2018). Left-liver adult-to-adult living donor liver transplantation: can it be improved? A retrospective multicenter European study. Ann Surg.

[3] Schnitzbauer AA, Lang SA, Goessmann H, Nadalin S, Baumgart J, Farkas SA (2012). Right portal vein ligation combined with in situ splitting induces rapid left lateral liver lobe hypertrophy enabling 2-staged extended right hepatic resection in small-for-size settings. Ann Surg.

[4] Ekman M, Fjälling M, Holmberg S, Person H (1992). IODIDA clearance rate: a method for measuring hepatocyte uptake function. Transplant Proc.

[5] de Graaf W, van Lienden KP, Dinant S, Roelofs JJTH, Busch ORC, Gouma DJ (2010). Assessment of future remnant liver function using hepatobiliary scintigraphy in patients undergoing major liver resection. J Gastrointest Surg.

[6] Mosteller RD (1987). Simplified calculation of body-surface area. N Engl J Med.

[7] Scatton O, Cauchy F, Conti F, Perdigao F, Massault PP, Goumard C (2016). Two-stage liver transplantation using auxiliary laparoscopically harvested grafts in adults: emphasizing the concept of "hypersmall graft nursing". Clin Res Hepatol Gastroenterol.

[8] Königsrainer A, Templin S, Capobianco I, Königsrainer I, Bitzer M, Zender L (2019). Paradigm shift in the management of irresectable colorectal liver metastases: living donor auxiliary partial orthotopic liver transplantation in combination with two-stage hepatectomy (LD-RAPID). Ann Surg.

[9] Nadalin S, Königsrainer A, Capobianco I, Settmacher U, Rauchfuss F (2019). Auxiliary living donor liver transplantation combined with two-stage hepatectomy for unresectable colorectal liver metastases. Curr Opin Organ Transplant.

[10] Allard M-A, Adam R, Bucur P-O, Termos S, Cunha AS, Bismuth H (2013). Posthepatectomy portal vein pressure predicts liver failure and mortality after major liver resection on noncirrhotic liver. Ann Surg.

[11] Botha JF, Langnas AN, Campos BD, Grant WJ, Freise CE, Ascher NL (2010). Left lobe adult-to-adult living donor liver transplantation: small grafts and hemiportocaval shunts in the prevention of small-for-size syndrome. Liver Transpl.

[12] Sudhindran S, Menon RN, Balakrishnan D (2012). Challenges and outcome of left-lobe liver transplants in adult living donor liver transplants. J Clin Exp Hepatol.

[13] Umeda Y, Yagi T, Sadamori H, Matsukawa H, Matsuda H, Shinoura S (2008). Effects of prophylactic splenic artery modulation on portal overperfusion and liver regeneration in small-for-size graft. Transplantation.

